# Immunotherapy in Cancer Management: A Literature Review of Clinical Efficacy of Pembrolizumab in the Non-small Cell Lung Cancer Treatment

**DOI:** 10.34172/apb.2023.007

**Published:** 2021-10-10

**Authors:** Luísa Biscaglia Miquelotti, Marcel Henrique Marcondes Sari, Luana Mota Ferreira

**Affiliations:** ^1^Departamento de Farmácia Industrial, Curso de Farmácia, Centro de Ciências da Saúde, Universidade Federal de Santa Maria, Santa Maria, Brazil.; ^2^Curso de Farmácia, Instituto de Desenvolvimento Educacional de Passo Fundo - UNIDEAU, Passo Fundo, Brazil.; ^3^Programa de Pós-graduação em Ciências Farmacêuticas, Centro de Ciências da Saúde, Universidade Federal de Santa Maria, Santa Maria, Brazil.

**Keywords:** Immunotherapy, Non-small cell lung cancer, Pembrolizumab, Anti-CTL4, Anti-PD-1

## Abstract

***Purpose:*** Cancer is a global public health problem that affects millions of people every year and the immunotherapy has been a promising alternative for its treatment. The aim of this study was to gather data concerning the efficacy and safety of immunotherapy in the treatment of non-small cell lung cancer (NSCLC), emphasizing pembrolizumb, a humanized antibody. This study also reports the role of immunotherapy in cancer treatments, contemplating the anti-CTLA4, anti-PD-L1 and anti PD-1 action in lymphocyte T cells.

***Methods:*** A bibliographic review was performed using Pubmed, SCIELO and SCOPUS databases, screening the scientific studies published within the last 5 years.

***Results:*** Seven clinical trials were selected to discuss the benefits of pembrolizumab as NSCLC therapy in untreated and previously treated patients, considering or not the tumor proportion score (TPS). It was found that NSCLC occurs with great frequency in Brazil and worldwide, presenting a poor prognosis due to its late diagnosis in most cases. Immunotherapy is a promising treatment strategy for NSCLC because its benefits overcome its risks compared to other therapies. Besides, the studies evidenced the efficiency of pembrolizumab as monotherapy or in association whit chemotherapy, in the first or second line of treatment and, additionally, patient’s whit TPS ≥ 50% seem to have a greater benefit from the treatment.

***Conclusion:*** The data collected herein showed that pembrolizumab is a very promising, effective, and safe treatment option against NSCLC. Lastly, it is important to highlight the relevance of review’s studies, since they are easy-to-read materials, collecting relevant information on a subject.

## Introduction

 Lung cancer is a type of tumor that has a high-frequency rate worldwide. Recent data demonstrated that it is the second most common cancer in men and women and ranks the first most deadly cancer to both female and male sex in the United States.^1^ It was estimated that 90% of cases are diagnosed due to smoking habits and passive exposure to cigarette smoke.^2^ Non-small cell lung cancer (NSCLC) corresponds to around 80% of the lung cancer cases and its main subtypes are adenocarcinoma and squamous cell carcinoma.^3,4^ Most cases are detected in advanced stages and are treated using chemotherapy or immunotherapies, such as pembrolizumab.^5-8^

 Immunotherapy has its origin in the experiment of the surgeon and oncologist William Coley, who developed the applied bacteria-derived toxins methodology to treat a certain type of cancer in the 19^th^ century and is now becoming relevant in the management of other tumor types.^9^ The treatment is based on the reactivation of the human immune response to recognize and kill cancer cells, which normally use different mechanisms to stealth from the destruction triggered by the immunological system.^7,10^

 The most used types of immunotherapies are specific immunological stimulants, vaccines, and immunological checkpoint inhibitors. An important checkpoint is the cytotoxic T lymphocyte (CTLA-4), which is inhibited by some drugs or even anti-cell death programmed-1 (PD-1).^7,11^ Pembrolizumab is an anti-PD-1 monoclonal antibody that has been approved for the treatment of several types of tumors such as melanoma, gastric cancer, classic Hodgkin’s lymphoma and NSCLC.^12^ Its action occurs by inhibiting the PD-1 receptor binding to PD-L1 and PD-L2 ligands, which provides T cells to remain in the active state, prolonging the functioning of T lymphocytes focused on the elimination of tumor cells.^13^ Its use for the treatment of NSCLC was approved by the FDA in October 2016 and is gaining more space in the treatment of this disease due to its lower toxicity compared to chemotherapies.^14^

 Advances in immunotherapy are becoming frequent and its application for cancer treatment has been presenting some advantages in comparison to the conventional protocol. Among them, the monoclonal antibody pembrolizumab has been shown to a great clinical efficacy by improving the survival rate of patients affected by the disease. Therefore, the current study sought to perform a literature survey regarding the important advances in the treatment of NSCLC by using pembrolizumab.

## Methods

The present study contemplates a narrative literature review, which enables greater familiarity with a predetermined subject. The review was conducted based on the PRISMA guidelines, according to the following steps^15^: (I) identification of the research theme and guiding question elaboration; (II) inclusion and exclusion criteria definition; (III) selection of articles by reading the title and summary. Lastly, after a detailed revision of the selected studies, (IV) it was collected the main data as overall survival, progression-free survival, and tumor proportion score (TPS) in relation to PD-L1 status to further evaluate the results and produce the review.

###  Search strategy and selection criteria

 This research addresses and describes the immunotherapy role in cancer treatments, mainly in patients with NSCLC emphasizing pembrolizumab. The scientific data were extracted from original and review articles, randomized clinical trials, cohort studies, and books that were published in the last five years. Electronic research was carried out on the PubMed, SCIELO, and SCOPUS databases. The terms “immunotherapy”, “non-small cell lung cancer” and “pembrolizumab” were used as keywords for scientific literature screening as well as their variants in Portuguese and Spanish. Publications that did not fit the theme delimitation, opinion or reflection articles, and editorials were excluded.

###  Data synthesis

 At first, a theoretical approach on the mechanism of immunotherapy in cancer treatment was elaborated. Following, a survey of studies that applied the pembrolizumab in the treatment of NSCLC was carried out. The data were expressed in Table 1, providing the drug doses applied, publication year, type of study, and the specification of the tumor investigated. The main data collected from the studies were tabulated for better visualization of essential findings, such as the overall survival, progression-free survival, and TPS in relation to PD-L1 status.

**Table 1:** Selected studies on pembrolizumab for the treatment of NSCLC

**Study**	**Doses**	**Therapy design**	**Study design**	**Tumor Specification**	**Ref.**
KEYNOTE-001	2 mg or 10 mg/kg/3 weeks	Monotherapy	Clinical trial	NSCLC, previously treated and untreated	^16^
KEYNOTE-010	2 mg or 10 mg/kg/3 weeks	Monotherapy X Docetaxel	Randomized study	Advanced NSCLC previously treated	^17^
KEYNOTE-024	200 mg/3 weeks	Monotherapy X Platinum-based chemotherapy	Randomized open study	Previously untreated advanced NSCLC, PD-L1 TPS ≥ 50%	^18^
KEYNOTE-189	200 mg/3 weeks	Pembrolizumab *plus* chemotherapy	Double-blind clinical trial	Previously Untreated Non-Squamous Metastatic NSCLC	^19^
KEYNOTE-407	200 mg/3 weeks	Pembrolizumab *plus* chemotherapy	Double-blind clinical trial	Previously untreated metastatic squamous NSCLC	^20^
KEYNOTE-042	200 mg/3 weeks	Monotherapy X Platinum-based chemotherapy	Randomized open study	Previously Untreated NSCLC PD-L1 TPS ≥ 1%	^21^
Subgroup KEYNOTE-024 Japan	200 mg/3 weeks	Monotherapy X Platinum-based chemotherapy	Randomized open study	Previously untreated metastatic NSCLC PD-L1 TPS ≥ 50%	^22^

## Results and Discussion

###  Immunotherapy

 Immunotherapy consists of using agents to reactivate the host’s immune system to remove tumor cells. Different from chemotherapy, immunotherapy restricts the damage to altered cells and saves the healthy ones due to the highly specific immunological response.^23,24^

 The interaction between cancer and the immune system is explained by immunoediting, a process constituted by three stages: the first is the elimination step, related to the immunosurveillance, which provides the detection and death of malignant cells mediated by the immune system; the second phase is equilibrium, in which the cells that were not eliminated during the first phase coexist with the immune system, which only prevents the tumor progression without eliminating the altered tissue and can last for years; the last one is escape, described by uncontrollably growing of cancer cells due to the immune system overload. The escape stage occurs through several mechanisms including suppression of the immune response by the tumor cells and their increased resistance.^10,25,26^

 Due to the many escape mechanisms and the complexity of tumor cells-induced immunosuppression process, there are a variety of immunotherapies available for certain types of cancer. Currently, some specific immunotherapeutic classes are receiving special attention from regulatory entities, such as the adoptive cell transfer therapy (ACT) and immune checkpoint inhibitors (ICI).^23^ ICI acts in cytotoxic T lymphocyte via the lymphocyte-associated protein 4 (CTLA-4) pathway as well as via programmed cell death PD-1 and its ligand -1 (PD-L1) pathway.^10^

**Figure 1:**
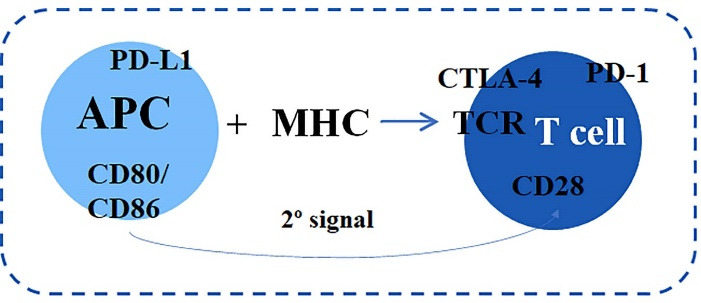
Initial step for the T lymphocyte activation.^10,25,26^

 The cytotoxic T cells activation depends on the antigen-presenting cells (APC), which recognize the foreign molecule and associated with the major histocompatibility complex present it to the T lymphocyte receptor (TCR) (Figure 1). Following, the second signal occurs by co-stimulatory activating, such as the T lymphocyte CD28 receptor, through CD80/CD86 ligands binding on APCs. On T lymphocytes surface, co-inhibitory molecules such as the CTLA-4 and PD-1/ PD-L1 receptor are also expressed.^9,10^

**Figure 2:**
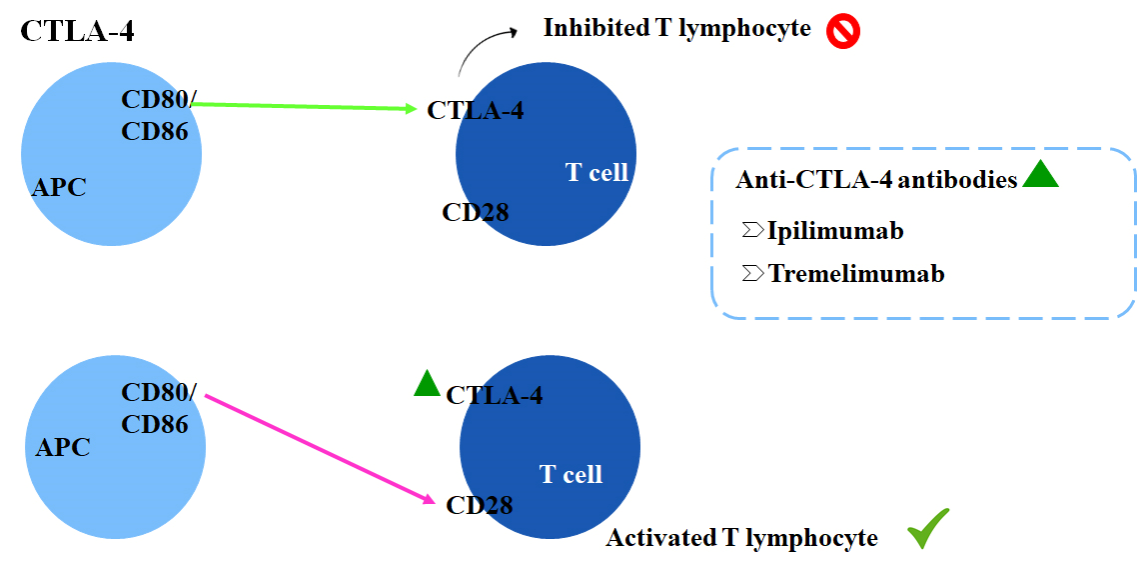
Inhibition of T lymphocytes caused by CD80/CD86 binding to CTLA-4 and mechanism of action of anti-CTLA-4 monoclonal antibodies.^11,27,28^

 CTLA-4 is a receptor expressed on T cells surface and acts as a negative regulator of T cells function. It has structural similarity with CD28and superior affinity for the ligands CD80/CD86, competing against CD28.^27,28^ The monoclonal antibodies targeting CTLA-4 (anti-CTLA4) block specifically this receptor and prevent the binding to CD80/CD86, which enables the interaction of co-stimulator and prolongs T cell activation as well as the antitumor response, as demonstrated in Figure 2.^28^

**Figure 3:**
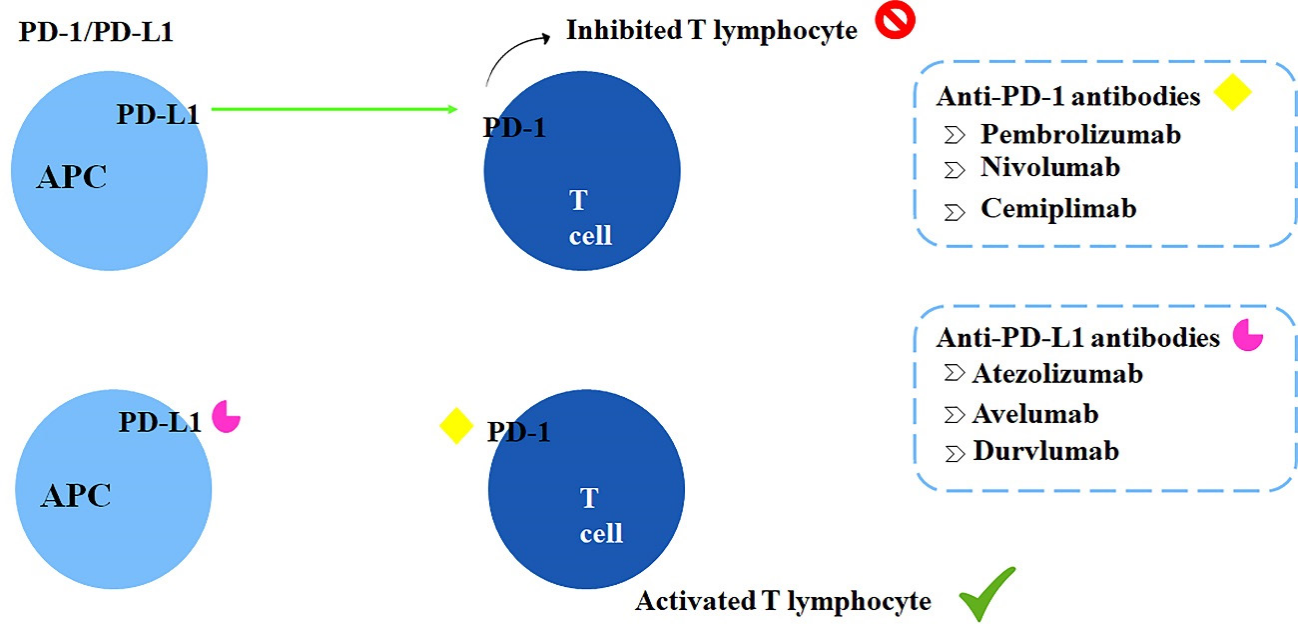
Inhibition of T lymphocytes caused by PD-L1 binding to PD-1 and mechanism of action of anti-PD-1 and anti-PD-L1 monoclonal antibodies.^11,24,25^

 PD-1 is also a co-inhibitory receptor expressed on the surface of some immune cells, such as B lymphocytes, NK cells, and activated T lymphocytes. Importantly, the malignant cells express the PD-L1 ligand on their surface, which acts as a potential activator of PD-1 receptors and induces T cells anergy.^11,24^ Anti-PD-1 antibodies prevent the PD-1 receptor activation, maintaining T cells functioning and prolonging the immune attack against malignant cells; the mechanism of action of these strategies are shown in Figure 3.^25^ Interactions between CTLA-4 and B7(CD80/CD86) as well as PD-1 and its ligand PD-L1 induce lymphocyte silence, creating immunological tolerance. Tumor cells use this mechanism to avoid destruction and elimination. Immune checkpoint inhibitors block CTLA-4 and the PD-1/PD-L1 axis maintaining lymphocytes reactive against tumors.

 However, despite the superior specificity and lower side effects to healthy cells in comparison to chemotherapeutic agents, immunotherapy may cause adverse reactions. They are classified according to their severity, presenting mild toxicity (grade 1) or even fatal toxicity (grade 4).^10^ The most common adverse events are related to cutaneous toxicity and affect approximately 35% and 44% of patients who receive anti-PD-1 or anti-CTLA-4 therapy, respectively. In addition, pruritus, skin rashes, erythema, and some more severe skin issues, such as Stevens-Johnson syndrome; other frequent events that may occur during treatment are diarrhea, colitis, fatigue, nausea, and pneumonitis.^10,29^ Additionally, a study demonstrated that the monoclonal antibody BMS-936559, an anti-PD-L1 therapy, induced myasthenia gravis as well as myocarditis, vasculitis and anemia, and so on.^10,29^

 The already available immunotherapeutic drugs are ipilimumab and tremelimumab, both anti-CTLA-4 antibodies. Pembrolizumab, nivolumab, and cemiplimab, representing the anti-PD-1 antibodies class. Lastly, the anti-PD-L1 antibodies atezolizumab, avelumab, and durvalumab.^11^ In 2016, pembrolizumab was approved by the FDA and 2018 in Brazil by ANVISA as an immunotherapeutic drug for NSCLC in the first- and second-line treatment. Therefore, based on the recent advances concerning pembrolizumab efficacy for cancer management, this review focused on a critical evaluation of the already published clinical trials performed using pembrolizumab for treating NSCLC.

###  Pembrolizumab

 Pembrolizumab (Keytruda^®^) is a humanized IgG4 monoclonal antibody that acts selectively against the PD-1 receptor. Its clinical use is indicated for treating several types of tumors such as melanomas, NSCLC, urothelial carcinoma, gastric cancer, Hodgkin’s lymphoma classic, renal cell carcinoma, head and neck cancer, and esophageal cancer.^12^ Of particular importance, no cytotoxic effect is observed by the antibody binding with PD-1 because it does not include Fc receptors, avoiding complement system activation.^13,30^

 Some types of tumor cells express the PD-L1 ligand, which can induce immune cells inactivation, mitigating T lymphocytes destructive responses against the altered cells. Pembrolizumab acts by blocking PD-1 receptor, preventing its binding to PD-L1, and maintaining the T lymphocytes reactivity against neoplastic cells.^13^

 The antibody drug is commercially available as an injectable formulation suitable for intravenous administration, providing an immediate and complete bioavailability of the dosage.^12^ The drug presents a low rate of protein-binding and a half-life of around 25 days. Its catabolism occurs through non-specific pathways and its metabolization does not favor its clearance, which has a geometric mean of approximately 0.2 L/day.^12^

 According to some clinical studies, pembrolizumab seemed to be safe at doses of 1-10 mg/kg every 2 weeks. In pathological conditions that required superior dosages used 2 mg/kg every 3 weeks, which is the usual approved dose of the drug. The steady-state concentrations are reached within 19 days by applying this repeated dosing interval.^12^ Additionally, studies revealed that parameters such as body weight, age, sex, tumor type, and burden as well as renal and liver failure did not impact exposure to pembrolizumab. Based on these findings, a fixed dose of 200 mg of the drug every 3 weeks was approved.^12,13^

 Despite that immunotherapy could restore antitumor immunity there are some immune-related adverse events, which are often distinctly different from the classical chemotherapy-related toxicities.^31^ Pembrolizumab is a safe and tolerable drug, but its adverse effects may appear later, even after the end of the treatment.^12^ The most common adverse events are fatigue, itching, diarrhea, and some skin rashes. Regarding the skin issues, such cutaneous manifestations could be the product of more potent immune system activation owing to immune-modulatory drugs.^32^ Some fewer common effects that can occur are nausea, headache, asthenia, myalgia, loss of appetite, and hypothyroidism. Although rarely documented, pneumonitis is the most serious adverse event and its clinical outcomes vary from pulmonary infiltration to severe pneumonia.^12,30^

###  Pembrolizumab and non-small cell lung cancer

 Pembrolizumab has been approved by the FDA as a first-line treatment for NSCLC. Its approval was granted due to this type of tumor cells express PD-L1, which is an essential characteristic for the treatment.^14^ The screening of scientific literature demonstrated the benefits and efficacy of treating NSCLC patients with pembrolizumab. Data revealed different dosages and conditions, such as the TPS, which is the degree PD-L1 expression, and whether or not there was a previous treatment, as shown in Table 1.

 The first study investigated the safety, adverse effect profile, and antitumor action of pembrolizumab as monotherapy, named KEYNOTE-001 clinical trial. Additionally, the link between a greater benefit of pembrolizumab and the PD-L1 tumor expression level in patient was defined. In the study, validation groups were composed of untreated and previously treated patients, without the specification of received treatment. Patients were treated with a pembrolizumab dose of 2 or 10 mg/kg every 3 weeks or 10 mg/kg every 2 weeks for 30 minutes.^16^ The most common adverse effects were fatigue, itching and decreased appetite, which occurred in 70.9% of patients. More serious adverse events were observed in only 9.5% of patients, such as hypothyroidism (6.9%) and pneumonitis (3.6%). Previously treated patients presented a response rate of 18.0% and 24.8% for previously untreated patients. In relation to overall survival, patients that already received any treatment presented a mean of 9.3% against 16.2% to untreated patients. Concerning the level of tumor expression, the response rate for patients with TPS ≥ 50% was 45.2%, and both groups with TPS from 1 to 49% and TPS < 1 did not exceed this value. Thus, TPS ≥ 50% was associated with longer survival as well as superior significant response rates. Lastly, the lowest dosage applied in the study was defined as the recommended dose because all those tested presented similar efficacy and safety profiles.^16^

 KEYNOTE-024 is a study that was carried out to verify the benefits of pembrolizumab over others therapies. A total of 305 advanced NSCLC patients that were not previously treated with PD-L1 TPS ≥ 50% were divided into two groups. Each group received a different protocol of treatment, as follows: (a) pembrolizumab at a dose of 200 mg every 3 weeks for 35 cycles; (b) an association of drugs during 4 to 6 cycles, such as carboplatin plus pemetrexed, cisplatin plus gemcitabine, cisplatin plus pemetrexed, carboplatin plus gemcitabine or carboplatin plus paclitaxel (majority choice). The mean duration of treatment was 7 months for the group (a), two-fold higher than group (b) (3.5 months). The progression-free survival rate was 62.1% in group (a) and 50.3% in the chemotherapy group (b). The objective response rate (ORT) was almost two-fold higher in the group (a), about 44.8% versus 27.8% in the chemotherapy group (b). Adverse events occurred more frequently in the group that used platinum-based chemotherapy, occurring in 90.0% of patients and 73.4% in the other group; Grade 3,4, and 5 events that occurred in the pembrolizumab group were diarrhea and pneumonitis (2.6% of patients) and anemia, neutropenia, thrombocytopenia, fatigue, and decreased appetite on chemotherapy treatment.^18^ It was found that pembrolizumab presents a superior efficacy when compared to platinum-based chemotherapy as it improves the overall and progression-free survival rate of patients, as well as a higher and longer-lasting response rate. These benefits overcome the risks of its already known adverse events, which were less frequent than in the chemotherapy group (b) even considering longer exposure periods.^12,14,17^

 In the KEYNOTE-010 study, the pembrolizumab treatment was tested in patients that previously received platinum-based chemotherapy, with PD-L1 TPS ≥ 1% or PD-L1 ≥ 50%. A total of 1034 patients were submitted to different protocols of treatment, which consisted of doses at 2 mg/kg (345 patients) or 10 mg/kg (346 patients) for 30 minutes every 3 weeks (total of 35 cycles), compared to docetaxel 75 mg/m^2^ for 1 hour every 3 weeks (343 patients). Over the study, 521 patients died, totaling 50% of the group with pembrolizumab 2 mg/kg, 45% of the drug at a dose of 10 mg/kg and 56% of the docetaxel group. Data regarding the overall survival and progression-free survival rates (Table 2) and the survival rates with tumor PD-L1 expression ≥ 50% (Table 3) demonstrated that pembrolizumab was superior in all abovementioned parameters in comparison to patients with higher expression of PD-L1. Grade 3-5 adverse events occurred in 13% of patients who received the lowest dose of antibody, 16% that received 10 mg/kg, and 35% of patients treated with docetaxel. Both groups treated with pembrolizumab increased in the overall survival rate and lowered adverse events.^17^

**Table 2:** Overall survival rate (months) and progression-free survival at different dosages and therapies

**Treatment**	**Overall survival**	**Progression free survival**
Pembrolizumab 2 mg/kg	10.4	3.9
Pembrolizumab 10 mg/Kg	17.3	5.2
Docetaxel	8.5	4.0

**Table 3:** Overall survival rate (months) and progression-free survival at different dosages and therapies with TPS PD-L1 ≥ 50%

**Treatment**	**Overall survival**	**Progression free survival**
Pembrolizumab 2 mg/kg	14.9	5.0
Pembrolizumab 10 mg/kg	12.7	4.0
Docetaxel	8.2	4.1

 In the KEYNOTE-042 study, patients with PD-L1 TPS ≥ 1% without prior treatment received pembrolizumab at a dose of 200 mg every 3 weeks or a selected carboplatin chemotherapy, similarly to KEYNOTE study -024. The aim of this study was to evaluate the treatment efficacy in patients with TPS ≥ 1% as well as to expand the number of volunteers (305 patients – 024 and 1274 patients - 042). In this clinical trial, half of the patients received pembrolizumab and the other half the chemotherapy. Treatment with platinum chemotherapy was associated with paclitaxel 200 mg/m^2^ or pemetrexed 500 mg/m^2^.^21^ Patients were separated according to their TPS and evaluated based on survival rate (Table 4). Adverse events occurred in 63% of patients in the pembrolizumab group, the most common was hypothyroidism (11%). In the chemotherapy group, 90% of volunteers presented adverse reactions and anemia (37%) was the most frequent one. Therefore, this study showed that pembrolizumab monotherapy improves overall survival and fewer adverse events. Besides, the study also demonstrated that the pembrolizumab advantages seem to increase with PD-L1 expression in patients.

**Table 4:** Overall survival rate (months) of patients with different TPS PD-L1

**TPS**	**Pembrolizumab**	**Chemotherapy**
≥ 50 %	20.0	12.2
≥ 20 %	17.7	13.0
≥ 1 %	16.7	12.1

 The KEYNOTE-189 study evaluated patients with non-squamous metastatic NSCLC without previous treatment that received pemetrexed 500 mg/m^2^, a platinum-based drug (cisplatin 75 mg/m^2^ or carboplatin 5 mg/mL/min) and 200 mg of pembrolizumab or placebo every 3 weeks for 4 cycles, followed by pembrolizumab (410 patients) or placebo (206 patients) until complete 35 cycles. After monitoring the volunteers for 10.5 months, the data revealed an overall survival rate of 69.2% for the protocol of chemotherapy + pembrolizumab and 49.4% for placebo + chemotherapy. Progression-free survival was 8.8 months against 4.9, respectively and the response rate was higher in the pembrolizumab group with 47.6% versus 18.9% for placebo. Adverse events classified as grade 3 or higher occurred in a similar number of patients to pembrolizumab (67.2%) and placebo (65.8%) as well as the number of deaths (6.7 % and 5.9 %, respectively). Acute kidney injury occurred in 5.2% of patients treated with pembrolizumab but was associated with pemetrexed and platinum.^19^

 In KEYNOTE-407, previously untreated metastatic squamous NSCLC patients were divided into pembrolizumab group (total of 278 patients – 200 mg) and placebo group (total of 281patients), receiving the treatments during 35 cycles. Until the 4^th^ cycle, both groups received carboplatin and paclitaxel (200 mg/m^2^) or nab-paclitaxel (100 mg/m^2^). The overall survival rate of patients with different levels of TPS demonstrated a significant improvement for patients who underwent a treatment combined with the antibody (Table 5). In addition, the progression-free survival rate was 6.4 months in the pembrolizumab group and 4.8 months in the placebo one, which was an improvement observed in all TPSs. Grade 3 or higher adverse events were slightly more frequent in the pembrolizumab-treated group than in the placebo group, 69.8% versus 68.2%, respectively. The adverse effects led to the treatment discontinuation for a portion of the volunteers (13.3% to the pembrolizumab group and 6.40% to the placebo group). Despite this, the treatment with pembrolizumab proved to be more efficient because it increased the overall survival and progression-free survival rates as well as a superior ORT (57.9%) in comparison to the placebo group (38.4%).^20^

**Table 5:** Overall survival rate of patients with different TPS PD-L1

**TPS**	**Pembrolizumab**	**Placebo**
≥ 50 %	63.4 %	51.0 %
1 a 49	65.9 %	50.0 %
< 1 %	64.2 %	43.3 %

 Corroborating with these data, in a study, carried out in Japan, the benefits of pembrolizumab were confirmed. The drug provided a higher survival rate an ORT of 62% (200 mg every 3 weeks, a total of 35 cycles) over an average exposure time of 13.1 months. In the course of the treatment, no safety problems were observed, ensuring the tolerability of the drug in the patients. Comparatively, platinum-based chemotherapy (4 to 6 cycles) presented an ORT of 26%. During the study, the efficacy and safety of the drug were proven in relation to platinum-based chemotherapy, supporting pembrolizumab monotherapy as a first-line treatment for PD-L1 positive NSCLC.^22^

 Pembrolizumab was shown to be a first-line therapy for the treatment of NSCLC in both previously treated and untreated patients. The studies demonstrated a higher survival rate for patients with tumors that showed higher expression of PD-L1. In addition, pembrolizumab used in monotherapy has shown more promise than conventional chemotherapy, increasing overall survival and progression free survival. In addition, there was greater safety due to the lower toxicity of the treatment and a better quality of life for patients compared to other therapies such as chemotherapy, which is highly associated with serious adverse effects and are less beneficial to patients.

## Conclusion

 NSCLC is an aggressive disease that presents high rates of metastasis and poor prognosis due to late diagnosis. Despite the efficacy of chemotherapy agents, the survival rates of patients are low and several toxic issues are triggered by their use, causing therapy dropout. In this sense, the development and approval of immunotherapies were of considerable importance because they offer treatments with similar efficacy to other existing treatments associated with several advantages, such as the higher survival rate and fewer adverse effects, improving the life quality of patients.

 The current study provided important data concerning the incidence and aggressiveness of NSCLC as well as the importance of immunotherapy with pembrolizumab in the treatment for a better disease prognosis. Furthermore, the development of bibliographic reviews is of great relevance, because they are easy-to-read materials, bringing the synthesis of scientific evidence about a specific subject, which allows health professionals to choose an effective and safe therapy for patients.

## Acknowledgments

 We would like to thank Veronica Ferrari Cervi for kindly reviewing the English language.

## Author Contributions

**Conceptualization:** Luísa Biscaglia Miquelotti; Marcel Henrique Marcondes Sari; Luana Mota Ferreira.

**Data curation: **Luísa Biscaglia Miquelotti.

**Formal Analysis:** Luísa Biscaglia Miquelotti; Marcel Henrique Marcondes Sari; Luana Mota Ferreira.

**Writing – original draft:** Luísa Biscaglia Miquelotti; Marcel Henrique Marcondes Sari; Luana Mota Ferreira.

**Writing – review & editing:** Marcel Henrique Marcondes Sari; Luana Mota Ferreira.

## Conflicts of Interest

 The authors declare no conflicts of interest.

## Ethical Issues

 Not applicable.
